# A diagnostic scale for Alzheimer’s disease based on cerebrospinal fluid biomarker profiles

**DOI:** 10.1186/alzrt267

**Published:** 2014-06-26

**Authors:** Sylvain Lehmann, Julien Dumurgier, Susanna Schraen, David Wallon, Frédéric Blanc, Eloi Magnin, Stéphanie Bombois, Olivier Bousiges, Dominique Campion, Benjamin Cretin, Constance Delaby, Didier Hannequin, Barbara Jung, Jacques Hugon, Jean-Louis Laplanche, Carole Miguet-Alfonsi, Katell Peoc’h, Nathalie Philippi, Muriel Quillard-Muraine, Bernard Sablonnière, Jacques Touchon, Olivier Vercruysse, Claire Paquet, Florence Pasquier, Audrey Gabelle

**Affiliations:** 1 CHU de Montpellier and Université Montpellier I, IRMB, CCBHM, Laboratoire de Biochimie Protéomique Clinique, 80 Avenue Augustin Fliche, 34295 Montpellier, France; 2 Centre Mémoire Ressources Recherche Paris Nord Ile de France and Histologie et Biologie du Vieillissement, Groupe Hospitalier Saint-Louis Lariboisiere Fernand-Widal APHP, INSERM U942, Universite Paris Diderot, France; 3 Inserm U837 and Neurobiology Unit, Centre de Biologie-Pathologie, CHU, Universite Lille Nord de France, 59045 Lille, France; 4 Inserm U1079, University of Rouen, Department of Neurology and Laboratoire de biochimie, Rouen University Hospital, Rouen, France; 5 Centre Mémoire Ressources Recherche, Alsace; Department of Neurology, University Hospital of Strasbourg, Strasbourg, France; 6 2 ICube laboratory and FMTS (Fédération de Médecine Translationnelle de Strasbourg), team IMIS-Neurocrypto, University of Strasbourg and CNRS, Strasbourg, France; 7 Centre Mémoire Ressources Recherche Besancon Franche-Comté, Department of Neurology, CHU Besançon, Besançon, France; 8 Centre Mémoire Ressources Recherche, CHU, EA1040 Université Lille Nord de France, 59000 Lille, France; 9 Laboratoire de Biochimie et de Biologie Moléculaire, Hôpital de Hautepierre, Hôpitaux Universitaire de Strasbourg, Strasbourg, France; 10 Laboratoire de Biochimie Lariboisière-Fernand Widal Hospital, APHP, University Paris 7-Denis Diderot, University Paris Descartes, Paris, France; 11 Laboratoire de Biochimie, CHU de Besançon, Besançon, France; 12 Centre Mémoire Ressources Recherche Languedoc-Roussillon, CHU de Montpellier, Hôpital Gui de Chauliac, Montpellier, and Université Montpellier I, Montpellier, France; 13 Laboratoire de Neurosciences Cognitives et Adaptatives (LNCA), UMR7364, Université de Strasbourg-CNRS, Strasbourg, France

## Abstract

**Introduction:**

The relevance of the cerebrospinal fluid (CSF) biomarkers for the diagnosis of Alzheimer’s disease (AD) and related disorders is clearly established. However, the question remains on how to use these data, which are often heterogeneous (not all biomarkers being pathologic). The objective of this study is to propose to physicians in memory clinics a biologic scale of probabilities that the patient with cognitive impairments has an Alzheimer’s disease (AD) pathologic process.

**Methods:**

For that purpose, we took advantage of the multicenter data of our Paris-North, Lille, and Montpellier (PLM) study, which has emerged through the initial sharing of information from these memory centers. Different models combining the CSF levels of amyloid-β 42, tau, and p-tau(181) were tested to generate categories of patients with very low (<10%), low (<25%), high (>75%), and very high predictive values (>90%) for positive AD. In total, 1,273 patients (646 AD and 627 non-AD) from six independent memory-clinic cohorts were included.

**Results:**

A prediction model based on logistic regressions achieved a very good stratification of the population but had the disadvantages of needing mathematical optimization and being difficult to use in daily clinical practice. Remarkably, a simple and intuitive model based on the number (from zero to three) of three pathologic CSF biomarkers resulted in a very efficient predictive scale for AD in patients seen in memory clinics. The scale’s overall predictive value for AD for the different categories were as follows: class 0, 9.6% (95% confidence interval (CI), 6.0% to 13.2%); class 1, 24.7% (95% CI, 18.0% to 31.3%); class 2, 77.2% (95% CI, 67.8% to 86.5%); and class 3, 94.2% (95% CI, 90.7% to 97.7%). In addition, with this scale, significantly more patients were correctly classified than with the logistic regression. Its superiority in model performance was validated by the computation of the net reclassification index (NRI). The model was also validated in an independent multicenter dataset of 408 patients (213 AD and 195 non-AD).

**Conclusions:**

In conclusion, we defined a new scale that could be used to facilitate the interpretation and routine use of multivariate CSF data, as well as helping the stratification of patients in clinical research trials.

## Introduction

Intense research efforts have been conducted to develop and validate biomarkers to predict, detect, and follow up the progression of the disease or impact of potential new disease-modifying treatments of Alzheimer’s disease (AD). Brain imaging and biologic biomarkers are now included in the recommended AD diagnostic criteria [1-3]. Various scales or tools are available to physicians involved in AD research for better interpreting the positive or negative results of biomarkers for establishing a clinical diagnosis, such as the detection of hippocampal atrophy with the Scheltens’ scale [4] and the positivity of amyloid load on functional brain imaging via the Jack’s visual scale [5].

Although biologic cerebrospinal fluid (CSF) biomarkers are among the most studied and validated in clinical practice [6-8], no sort of “visual” scale concerning biologic biomarkers is available. Numerous studies did indeed show that AD patients display characteristic CSF changes with decreased levels of β-amyloid1-42 (Aβ42) and elevated levels of total tau protein (tau) and its phosphorylated form at threonine 181 (p-tau) [7,9,10]. Interestingly, these biomarkers are directly related to neuropathologic changes [11] present in the disease. Therefore, one would expect that the presence of these three biomarkers would be highly indicative of AD, whereas their absence would strongly disqualify this diagnosis. Other biomarkers represented by isoforms of Aβ (Aβ40, Aβ38) or linked to oxidative stress and inflammation [12,13] may also contribute to the diagnosis of AD, in particular when combined with tau or Aβ42. Apart from familial forms of AD, genetic profiles [14], in particular apolipoprotein E status, which represents the most prominent risk factor, is not yet used as a diagnostic tool.

So, today we can only rely routinely on the commonly measured CSF biomarkers Aβ42, tau, and p-tau to help us with AD diagnosis. In clinical practice, after control of preanalytic biases [15-17] and standardization of procedures [18-20], the performance of CSF biomarkers is satisfactory with a coupled sensitivity/specificity as high as 80% when used alone or combined. The combination of these biomarkers increases their performance, as demonstrated for Aβ42 and tau [21] or Aβ42 and p-tau [22,23]. In most AD biomarker studies, results are represented by the best sensitivity/specificity and area under the ROC curves (AUC). It is, however, difficult to use this information directly when biomarkers are being used in routine practice to help physicians with AD diagnosis [6-8]. We need a way to convert the variation of the three biomarkers into a probability scale for AD. A leading study from Spies *et al*. [24] proposed a logistic regression of logarithmic-transformed values of Aβ42 and p-tau values and sex defined classes of AD probability. Logistic regressions already demonstrated their relevance in differentiating AD patients from non-AD patients [7,23].

In the present study, we conducted an evaluation, based on multicenter data from our PLM cohort [25], of a simpler scale based solely on the numbers (from 0 to 3) of pathologic biomarkers (Aβ42, tau, and p-tau) identified. This intuitive scale was very efficient for generating categories of patients seen in memory clinics with a refined probability of AD, and it will represent a valuable tool for facilitating the interpretation and routine use of multivariate CSF data for stratifying patients into clinical research trials.

## Material and methods

### Study design and subjects

Patients (1,273) who had a lumbar puncture were recruited between January 2008 and December 2011 from the three initial PLM centers (Paris-North, Lille, and Montpellier) specialized in the care management of patients with cognitive disorders (Table 1). These centers used the same diagnostic procedures and criteria [25]. All patients had a thorough clinical examination, including biologic laboratory tests, neuropsychological evaluations, and brain imaging. Patients were classified into two groups: AD (as defined by the NINCDS-ADRDA criteria [26]), and non-AD (NAD) patients. NAD diagnosis (that is, frontotemporal lobar degeneration, semantic dementia, Lewy body and Parkinson diseases, progressive supranuclear palsy, amyotrophic lateral sclerosis, normal-pressure hydrocephalus, and psychiatric disorders), were defined by the commonly validated international criteria. Important for this study, the used diagnostic criteria for AD do not include CSF biomarkers (otherwise, that would bias the evaluation of the interest of the scale, which was based on biomarker values). Mild cognitive impairment, as well as phenotypes that were mixed with AD or might correspond to specific/early forms of AD, were excluded from the cohorts (mixed dementia, primary progressive aphasia, amyloid angiopathy). CSF was collected by using standardized collection, centrifugation, and storage conditions in different centers [25].

**Table 1:** Population demography and biomarker values

**Paris-1**			**Paris-2**			**Lille-1**			**Lille-2**			**Montpellier-1**			**Montpellier-2**			**RSB**		
**AD *n*** **= 118**	**Mean**	**SD**	**AD *n*** **= 41**	**Mean**	**SD**	**AD *n*** **= 118**	**Mean**	**SD**	**AD *n*** **= 73**	**Mean**	**SD**	**AD *n*** **= 129**	**Mean**	**SD**	**AD *n*** **= 142**	**Mean**	**SD**	**AD *n*** **= 213**	**Mean**	**SD**
Age	73.6	8.8	Age	70.9	8.9	Age	68.3	9	Age	67	9.5	Age	69.7	8.8	Age	71.1	10.1	Age	66.3	8.7
Aβ42	440	189	Aβ42	594	238	Aβ42	338	162	Aβ42	603	245	Aβ42	505	224	Aβ42	654	256	Aβ42	420	224
Tau	598	295	Tau	543	279	Tau	608	336	Tau	778	364	Tau	611	327	Tau	702	727	Tau	666	407
p-tau	99	40.4	p-tau	86	34	p-tau	98.1	46.9	p-tau	101.5	41.2	p-tau	85.9	40.2	p-tau	86	37.7	p-tau	92.4	38.1
MMSE	19.4	5.6	MMSE	18.7	6.7	MMSE	18.1	6.5	MMSE	19.6	6.3	MMSE	21.9	5.5	MMSE	20.7	7.4	MMSE	19.0	6.1
Sex (%M)	47%		sex (%M)	65%		sex (%M)	37%		sex (%M)	47%		sex (%M)	47%		sex (%M)	49%		sex (%M)	50%	
NAD *n* = 53	Mean	SD	NAD *n* = 68	Mean	SD	NAD *n* = 128	Mean	SD	NAD *n* = 51	Mean	SD	NAD *n* = 215	Mean	SD	NAD *n* = 147	Mean	SD	NAD *n* = 195	Mean	SD
Age	62.1	13.1	Age	67.4	11.1	Age	67.3	10.7	Age	64.6	10.7	Age	64.1	13.6	Age	63.4	13.6	Age	65.4	10.1
Aβ42	686	243	Aβ42	843	246	Aβ42	494	192	Aβ42	974	355	Aβ42	706	266	Aβ42	999	373	Aβ42	723	346
Tau	253	226	Tau	223	141	Tau	273	197	Tau	284	149	Tau	291	233	Tau	310	241	Tau	339	258
p-tau	48.6	23.1	p-tau	43.5	19.3	p-tau	52.6	28.7	p-tau	46.5	15.8	p-tau	44.8	23.7	p-tau	38.4	18.6	p-tau	49.3	25.8
MMSE	23	5.3	MMSE	23.6	4.9	MMSE	21.3	5.5	MMSE	21. 2	6	MMSE	20.7	7.1	MMSE	21.1	6.9	MMSE	21.0	6.1
Sex (%M)	53%		Sex (%M)	49%		sex (%M)	51%		sex (%M)	59%		sex (%M)	53%		sex (%M)	55%		sex (%M)	51%	

Mean and standard deviation of demographic data, CSF biomarker levels (Aβ42, tau, and p-tau) and Mini Mental Score (MMSE) for the patients with AD or NAD diagnosis in the different centers (Montpellier, Lille, Paris) in two independent cohorts that differed by their collection tubes (−1, −2) and in the three additional PLM centers: Rouen, Strasbourg, and Besançon (RSB).

A second set of data from 408 patients with the same clinical characteristics was generated from the three new PLM centers (Rouen, Strasbourg, and Besançon) also specialized in the care management of patients with cognitive disorders.

### CSF samples and assays

CSF Aβ42, tau, and p-tau concentrations were measured by using standardized commercially available INNOTEST sandwich ELISA, according to the manufacturer’s procedures (Fujirebio Europe NV, formerly Innogenetics NV). As the data were generated in the different centers through the routine activity of their laboratories, the lots of assay kits were variable within and in between laboratories. The quality of the results was ensured by the use of validated standard operating procedures and internal quality controls (QCs). The range of the QC coefficient of variation for Aβ42 across the different lots for the six laboratories was 5% to 11%. For tau and p-tau, the range was 8% to 14% and 6% to 14%, respectively. The use of external QC ensured also the quality of the results and the validity of the intersite data comparison [19]. The Paris, Lille, and Montpellier centers contributed with two sets of data labeled “-1” or “-2” generated from different collection tubes (for the −1 cohort in Montpellier, Greiner, catalog number 18 82 81; in Lille, Becton Dickinson, catalog number Falcon 35 2097; in Paris, CML, catalog number TC10PCS, and for the −2 cohorts: Sarstedt, catalog number 62.610.201) [20]. These two sets of data differed in their optimal cutoffs (≤506 and ≤834 for Aβ42; >343 and >340 for tau; >64 and >62 for p-tau; see Additional file 1) in relation with the preanalytic properties of the tube that affected mostly Aβ42 levels [27].

We pooled the data from the three additional PLM centers (Rouen, Strasbourg, and Besançon), which used standard cutoff biomarker values in relation to the type of collection tube used [20]. The lumbar puncture, the CSF biomarkers measurement, and the clinical examination (including the diagnosis) were part of standard care. This observational study on routine biologic analyses is not considered in France to be “biomedical research,” and it does not necessitate informed consent or ethical approval. Authorization for handling personal data has been granted by the French Data Protection Authority (CNIL) under the number 1709743 v0.

### Statistical analysis

Statistical analyses were computed with the MedCalc software (11.3). The logistic regression was performed by following a method similar to that of Spies *et al*. [24]. In brief, log-transformed CSF biomarkers and sex were entered with backward stepwise selection by using a significance level of 0.10. The logistic regression generates the coefficients of a formula to predict the logistic transformation of the probable presence of AD (p(AD)) as follows:

p(AD) = 1/(1 + 1 e – [intercept + score])

where score = coefficient × ln(Aβ42) + coefficient × ln(p-tau) + coefficient × Sex. Probability classes to discriminate between AD and non-AD patients were obtained by selecting the following ranges of p(AD): class 0, 0 to 0.1; class 1, 0.1 to 0.5; class 2, 0.5 to 0.9; and class 3, 0.9 to 1.0.

We did not perform the logistic regression analysis in one training population with the goal to apply the resulting classification to the other populations, but we rather recalculated the coefficients in each cohort to generate best-fitted models. Receiver operating characteristic (ROC) curves were used to represent sensitivity and specificity for AD detection. ROCs are generated from continuous diagnostic variables. The CSF biomarkers and their ratio are continuous, as well as the p(AD) values obtained after the logistic regression. Regarding the scales, we plotted the curve by using the four values 0, 1, 2, and 3 applied to the different samples.

To compare the classifications of patients, we used the net reclassification index (NRI) [28]. The NRI is based on reclassification constructed separately for participants with and without the event of interest (that is, AD or non-AD diagnosis), and quantifies the correct movement into classes, upward for events and downward for nonevents. At first, the following probabilities were calculated: p(up_AD) = (number of cases in which the class was moving up between two classifications of AD patients)/(number of AD patients); p(down_AD) = (number of cases in which the class was moving down between two classifications of AD patients)/(number of AD patients); p(up_NAD) = (number of cases in which the class was moving up between two classifications of NAD patients)/(number of NAD patients); p(down_NAD) = (number of cases in which the class was moving down between two classifications of NAD patients)/(number of NAD patients). We assumed that correctly classifying an AD patient was as important as correctly classifying an NAD patient, and therefore we computed the NRI by using the formula: NRI = (p(up_AD)-p(down_AD))-(p(up_NAD)-p(down_NAD)).

## Results

Demographics and biomarker values for the different cohorts are presented in Table 1. As expected, differences in individual biomarker concentrations between AD and NAD were apparent across all cohorts. ROC curves for Aβ42, tau, and p-tau were used to compute AUCs (Table 2) and optimal cutoff values (Additional file 1). AUCs were also calculated for Aβ42/tau and Aβ42/p-tau ratios, as well as the Aβ/tau index (IATI) [21] (Table 2). The Paris, Lille, and Montpellier PLM centers provided data from two independent cohorts (−1 and −2) that differed by the type of collection tube used (see Material and methods section). This explains the variations in cutoff values; the second tube having been selected for its low Aβ42 absorption [27], therefore resulting in higher cutoff values [20]. All patients of these three centers for whom samples were collected in tube 1 or 2 were also differentiated into two additional populations called PLM-1 and PLM-2. This was one way to evaluate the AD scale on multicenter cohorts after standardization of biologic and clinical practices. Data from these populations were used to compare predictions based on a logistic regression performed as shown by Spies *et al*. [24]. Interestingly, sex was never retained in the model used for our cohorts (Additional file 1). The models were very efficient in differentiating AD from non-AD (NAD) patients for all cohorts with AUCs close to 0.9 in most cases (Table 2).

**Table 2:** AUCs

**AUC**	**Paris-1**	**Paris-2**	**Lille-1**	**Lille-2**	**Mtp-1**	**Mtp-2**	**PLM-1**	**PLM-2**	**Mean**
**Aβ42**	0.81	0.768	0.778	0.826	0.747	0.778	0.772	0.787	0.783
**Tau**	0.898	0.921	0.869	0.905	0.84	0.852	0.86	0.88	0.878
**p-tau**	0.911	0.913	0.87	0.917	0.842	0.91	0.875	0.912	0.894
**IATI**	0.902	0.895	0.858	0.917	0.827	0.877	0.855	0.896	0.878
**Aβ42/tau**	0.913	0.921	0.88	0.927	0.849	0.882	0.874	0.905	0.894
**Aβ42/p-tau**	0.923	0.92	0.875	0.92	0.86	0.924	0.884	0.924	0.904
**Logi. reg.**	0.926	0.928	0.892	0.936	0.869	0.933	0.896	0.932	0.914
**Log. reg. scale**	0.917	0.931	0.872	0.927	0.838	0.918	0.876	0.919	0.900
**PLM scale**	0.94	0.931	0.887	0.919	0.863	0.933	0.883	0.924	0.910

Area under the ROC for the individual biomarkers Aβ42, tau and p-tau, the IATI [21] and the ratios Aβ42/tau and Aβ42/p-tau. The values of the p(AD) obtained after logistic regression were also used to calculate the AUC. The logistic regression values were separated into four classes (0 to 3), which values were then used, just as for the PLM scale, to calculate the AUCs.

**Figure 1:**
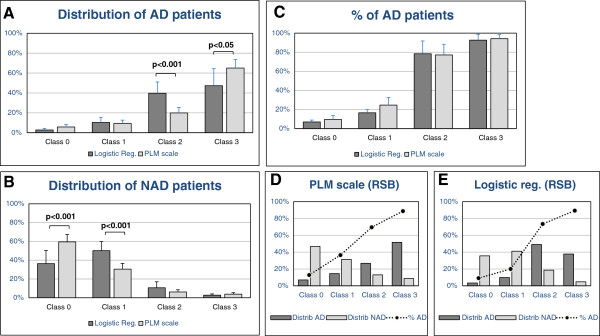
**Distribution and percentage of the AD and NAD patients between the different classes. (A, B)** The mean (±SD) was plotted of the distribution of the AD **(A)** and NAD **(B)** patients in the Paris, Lille, and Montpellier cohorts and across the four classes based on the logistic regression or the PLM scale. Significant differences (Student *t* test) were found between the percentage of AD patients in classes 2 and 3 **(A)**, as well as between the percentage of NAD patients in classes 0 and 1 **(B)**. **(C)** The mean (±SD) of the percentage of AD patients in each class was plotted. No significant difference was apparent. **(D, E)** The distribution of AD (dark gray bars) and NAD (light gray bars) patients in the populations of the Rouen, Strasbourg, and Besançon (RSB) centers. The percentage of AD in each class is also plotted (black dots linked a dotted line). Data obtained by using the PLM scale **(C)** show more AD patients in class 3 and NAD in class 0 than for the logistic regression **(E)**.

The distribution of AD and NAD patients was then evaluated according to four classes: between p(AD) 0 to 0.1 for class 0; 0.1 to 0.5 for class 1; 0.5 to 0.9 for class 2; and 0.9 to 1 for class 3 (Figure 1A,B and Additional file 2). The percentage of AD in each class was also computed (Figure 1C). As expected, in class 0, we found only around 10% of AD patients, whereas in class 3, this number was close to 90%, with slight variations related to differences in AD prevalence in these populations (Table 1).

The PLM scale composed of four classes was then designed, based on a very simple and intuitive rule: class 0, corresponding to no pathologic biomarkers (below cutoff for Aβ42, above cutoff for tau and p-tau); class 1, corresponding to one pathologic biomarker of three; class 2, corresponding to two pathologic biomarkers of three; and class 3, with all three biomarkers being pathologic. We calculated the sensitivity and specificity of this scale, with classes 0 and 1 grouped on one side, and classes 2 and 3, on the other side for NAD and AD diagnoses, respectively. Hence, a sample was defined as positive for AD if two or three biomarkers were pathologic (class 2 or 3). The performance of this simple rule was very high, as demonstrated by the sensitivity and specificity reached across the different cohorts (see Additional file 2). Moreover and interestingly, when all AUCs were compared (Table 2), the PLM scale was second best after the logistic regression.

**Figure 2:**
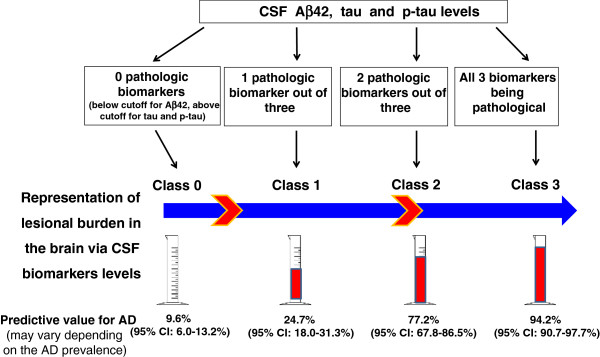
Graphic illustration of the AD scale.

Distributions of AD and NAD patients, as well as percentage of AD patients in each class, were then computed and plotted for the PLM scale (Figure 1A-C; Additional file 2). The percentage of AD patients in each class, which actually corresponded to the positive predictive value (PPV), that is, the percentage of true positives among all samples in the class, was compared between the two prediction models (Figure 1C). Predictive values were not significantly different between the logistic regression and PLM scale (Figure 1C). For the PLM scale, the average predictive values for AD in these cohorts were for class 0, 9.6% (95% CI, 6.0% to 13.2%); for class 1: 24.7% (95% CI, 18.0% to 31.3%), for class 2: 77.2% (95% CI, 67.8% to 86.5%), and for class 3, 94.2% (95% CI, 90.7% to 97.7%) (Figure 2).

The difference in patient distribution between the two prediction models was, however, significantly different, as the PLM scale had more AD patients in class 3 as well as more NAD patients in class 0 compared with the prediction obtained with the logistic regression (Figure 1A, B). This distribution of AD and NAD patients in different classes was also evaluated in an additional dataset from Rouen Strasbourg and Besançon (RSB) centers (Table 1). We used the standard cutoffs and logistic regression coefficients (Additional file 2) to compute and represent in Figure 1 (panels D and E) the distribution of AD and NAD patients across the different classes, as well as the percentage of AD patients in each class. With the PLM scale, profiles were comparable to those obtained before, with a better distribution of patients (more AD patients in class 3, and more NAD patients in class 0) and similar percentages of AD patients in each class.

To compare the classification resulting from the logistic regression and the PLM scales, we calculated the NRI (see Material and methods section) on the PLM-2 population. We observed that the PLM scale resulted in 23.25% more patients better classified (that is, having a higher scale value in the case of AD patients, and a lower value for non-AD patients). Similar results were obtained when the NRI was applied to the different cohorts (data not shown).

## Discussion

Diagnostic measures of AD include clinical observation, assessment of cognitive functions, including memory testing as well as advanced neuroimaging examinations such as magnetic resonance imaging (MRI) or positron emission tomography (PET), and CSF biomarkers [29]. In the present study, we validated the relevance of CSF biomarkers when used individually or within ratios [7,21-23]. Identifying one or several biomarkers or ratios as pathological is clinically relevant for the diagnosis of AD. However, the question remains as to how to use this element, which is often heterogeneous (not all biomarkers being pathological) in clinical practice. This complexity renders problematic the interpretation of biomarker results, and it differs between practitioners or centers.

Different rating systems or scales have being designed to help clinicians evaluate dementia. The clinical dementia rating (CDR), which is a 5-point scale based on cognitive and functional performances, does provide valuable clinical information [30]. The medial temporal lobe atrophy visual rating scale introduced by Scheltens *et al*. [31] is also a good example of a biomarker scale facilitating the diagnosis of AD. The challenge is to transform a multivariate panel into a score that predicts clinical outcomes. This was recently done to predict the conversion of mild cognitive impairment (MCI) patients into AD by using CSF Aß42, MRI, and PET data [32]. However, this model cannot be applied to our paradigm, which focuses on memory clinic cohorts suspected of having AD or other differential dementia diagnoses. Very relevant in this context is the leading work of Spies e*t al*., [24] who used a logistic regression on CSF biomarkers and sex to define a predictive model for AD. This model reached the highest performance for the diagnosis of AD, and it provides a continuous predictive scale made of 10 classes of AD probability (that is, none to 10%, 10% to 20%).

When we reproduced this approach for our datasets, the logistic regression did not retain sex in the models. The higher percentage of women in the AD population in the Spies cohort was probably responsible for this difference. In any case, the performance of the logistic regression was outstanding, as it resulted in the highest AUCs among individual or biomarker ratios (Table 2). To match the reports already used in clinical practice, we wanted to generate a simple rating with classes bearing very low (<10%), low (<25%), high (>75%), and very high predictive values (>90%) for AD. Four logistic regression classes with p(AD) ranging from 0 to 0.1, 0.1 to 0.5, 0.5 to 0.9, and 0.9 to 1 were therefore generated. This resulted in classes having an expected percentage of AD prediction (Figure 1C). We then compared this logistic regression approach with an intuitive classification based solely on the numbers (from 0 to 3) of pathological CSF biomarkers (Aß42, tau, and p-tau) (Figure 2).

The performance of this simple scale was evaluated in the different cohorts by using as a criterion of AD the presence of two or three pathologic biomarkers. Surprisingly, this simple rule resulted in high sensibility/specificity/AUCs. The predictive values of this “PLM scale” for the different classes were also similar to the ones obtained with the logistic regression (Figure 1C). Differences in the distribution of AD and NAD patients between classes were, however, noticeable, with significantly more patients correctly classified with the PLM scale. This apparent superiority of the PLM scale was validated by computing the NRI, which is a statistical tool designed to assess improvement in model performance offered by a new classification. This result could be explained by the “discontinuous” nature of the PLM scale, which is better suited to the four probability classes that we had targeted. Of note, a discrepancy between predicted and observed percentage of AD in regression classes was already observed by Spies *et al.*[24]. This triggered these authors to generate a second model with an extensive correction based on a second regression. This fine-tuning might overfit the models based on the individuals within a cohort, and it is therefore not adapted for the use of the scale in a predictive way for the classification of new patients in memory clinics.

In any case, the PLM scale outperformed the logistic regression and has the advantage of being used without complex mathematical adjustments. In addition, this classification gave a direct access to the percentage of discarding profiles, that is, AD patients with none of the biomarkers being positive (class 0), and conversely, NAD patients with three biomarkers being positive (class 3).

One of our study’s limits resides in using as reference the final clinical diagnosis, which, in the absence of neuropathologic information ,can sometimes remain uncertain. However, diagnoses were established by experienced multidisciplinary teams based on clinical, neuropsychological, and imaging data. The cross-validation of the results in the different cohorts also guarantees the reliability and relevance of our conclusions. Of note, the scale relies on the use of biomarker cutoffs that might differ in the different centers. The continuous improvement of the quality of the assays, thanks to the use of quality control, and the homogenization of preanalytics and operating procedure [15-20] may result in a scale that will use “unified” cutoffs. It is important to underline that the predictive value of the PLM scale is valid only to evaluate whether memory impairments or dementia is due to AD for patients seen in memory clinics. In other situations, and in particular for assessing the conversion from MCI to AD, the design and/or predictive value of the PLM scale should be revaluated.

We also must keep in mind that if we expect the distribution of AD and NAD patients across classes to be comparable between cohorts, the predicted percentage of AD patients in a given class depends itself on the prevalence of AD patients in the studied cohort. In the future, this scale might be improved by adding new variables and, in particular, the apolipoprotein E status, which today is not used routinely.

## Conclusion

We developed a simple and intuitive prediction model that demonstrated its relevance in identifying groups of patients with different predictive values for AD. This new scale can be used to facilitate the interpretation and routine use of multivariate CSF data and to stratify patients in clinical research trials.

## Abbreviations

AD: Alzheimer disease; AUC: area under curve; Aβ: amyloid β; CDR: clinical dementia rating; CI: confidence interval; CSF: cerebrospinal fluid; ELISA: enzyme-linked immunosorbent assay; MCI: mild cognitive impairment; MRI: magnetic resonance imaging; NAD: non-Alzheimer disease; NINCDS-ADRDA: Institute of Neurological and Communicative Disorders and Stroke and the Alzheimer Disease and Related Disorders Association; NRI: Net Reclassification Index; PET: positron emission tomography; PLM: Paris-North, Lille, and Montpellier; QC: quality control; ROC: receiver operating characteristic.

## Competing interests

All financial and material supports for this research are academic, and the authors declare no conflict of interest.

## Authors’ contributions

SL, JD, SS, CP, and AG: Study concept and design, analysis of the results, and drafting the manuscript. SL and JD: statistical analysis. SL, SS, KP, JLL, BJ, MQM, OB, CMA, NP, and BP: realization, follow-up and analysis of biologic measurements, and contribution to data interpretation and revision of the manuscript for important intellectual content. JD, DW, FB, EM, SB, DC, BC, DH, JH, JT, OV, CP, FP, and AG: clinical work, diagnosis confirmation, and contribution to data interpretation and revision of the manuscript for important intellectual content. All authors read and approved the final manuscript.

## Supplementary Material

Additional file 1 Cutoffs and logistic regression coefficients. Click here for file

Additional file 2 Logistic regression and PLM scale classification. Click here for file
