# Supplementary material for: A diagnostic scale for Alzheimer’s disease based on cerebrospinal fluid biomarker profiles
Source: Alzheimers Res Ther. 2014 Jun 26;6(3):38. doi: 10.1186/alzrt267 (PMC4255520; doi:10.1186/alzrt267)
Supplement: Additional file 1 — Cutoffs and logistic regression coefficients. [file alzrt267-S1.doc]

## Cutoffs and logistic regression coefficients

|  | **Optimal cutoffs** | | | **Logistic regression coefficients** | | | |
| --- | --- | --- | --- | --- | --- | --- | --- |
| Population | **Aβ42** | **tau** | **p-tau** | Intercept | ln(Ab42) | ln(p-tau) | Sex |
| **Paris-1** | <=494 | >282 | >63 | -4.2562 | -2.1841 | 4.5243 | 0 |
| **Paris-2** | <=633 | >336 | >62 | -3.0331 | -2.6424 | 4.92 | 0 |
| **Lille-1** | <=380 | >352 | >59 | -3.9445 | -1.6736 | 3.4383 | 0 |
| **Lille-2** | <=746 | >372 | >62 | -3.4918 | -1.9737 | 4.1233 | 0 |
| **Mtp-1** | <=572 | >351 | >63 | -1.7631 | -1.4972 | 2.6803 | 0 |
| **Mtp-2** | <=831 | >386 | >55 | -1.438 | -2.2435 | 4.,116 | 0 |
| **PLM-1** | <=506 | >343 | >64 | -3.512 | -1.5769 | 3.2586 | 0 |
| **PLM-2** | <=834 | >340 | >62 | -2.2675 | -2.2291 | 4.2275 | 0 |
| **RSB** | <=500 | >400 | >60 | -1.7475 | -1.2516 | 2.3688 | 0 |

Cutoffs corresponding to optimal values on the ROC curves (highest value of Youden index (sensitivity + specificity)) for Aβ42, tau and p-tau in the different populations. Coefficients obtained after logistic regression using ln(Aβ42), ln(p-tau) and Sex (see material and method section).
