# Supplementary material for: A diagnostic scale for Alzheimer’s disease based on cerebrospinal fluid biomarker profiles
Source: Alzheimers Res Ther. 2014 Jun 26;6(3):38. doi: 10.1186/alzrt267 (PMC4255520; doi:10.1186/alzrt267)
Supplement: Additional file 2 — Logistic regression and PLM scale classification. [file alzrt267-S2.doc]

## Logistic regression and PLM scale classification

| **Logistic Reg.** | **Criterion p(AD)>0.5** | | | **Distribution of AD** | | | | **Distribution of NAD** | | | | **% of AD in each class** | | | |
| --- | --- | --- | --- | --- | --- | --- | --- | --- | --- | --- | --- | --- | --- | --- | --- |
|  | **Sens.** | **Spe.** | **AUC** | **Class 0** | **Class 1** | **Class 2** | **Class 3** | **Class 0** | **Class 1** | **Class 2** | **Class 3** | **Class 0** | **Class 1** | **Class 2** | **Class 3** |
| Paris-1 | 94.1% | 81.1% | 0.917 | 0.8% | 5.1% | 26.3% | 67.8% | 24.5% | 56.6% | 13.2% | 5.7% | 7.1% | 16.7% | 81.6% | 96.4% |
| Paris-2 | 85.4% | 98.5% | 0.931 | 4.9% | 9.8% | 39.0% | 46.3% | 52.9% | 45.6% | 0.0% | 1.5% | 5.3% | 11.4% | 100.0% | 95.0% |
| Lille-1 | 87.4% | 77.5% | 0.872 | 0.7% | 11.9% | 43.4% | 44.1% | 14.7% | 62.7% | 20.6% | 2.0% | 6.3% | 21.0% | 74.7% | 96.9% |
| Lille-2 | 93.2% | 81.0% | 0.927 | 2.7% | 4.1% | 24.7% | 68.5% | 38.1% | 42.9% | 16.7% | 2.4% | 11.1% | 14.3% | 72.0% | 98.0% |
| Mtp-1 | 74.4% | 90.7% | 0.869 | 4.7% | 20.9% | 59.7% | 14.7% | 32.1% | 58.6% | 7.0% | 2.3% | 8.0% | 17.6% | 83.7% | 79.2% |
| Mtp-2 | 88.7% | 88.4% | 0.918 | 2.8% | 8.5% | 41.5% | 47.2% | 52.4% | 36.1% | 8.8% | 2.7% | 4.9% | 18.5% | 81.9% | 94.4% |
| PLM-1 | 84.6% | 85.1% | 0.876 | 2.1% | 13.3% | 47.4% | 37.2% | 28.4% | 56.8% | 11.6% | 3.2% | 6.1% | 13.0% | 52.4% | 89.1% |
| PLM-2 | 88.3% | 89.9% | 0.919 | 3.1% | 8.6% | 35.9% | 52.3% | 47.9% | 42.0% | 7.4% | 2.7% | 7.1% | 19.8% | 81.1% | 92.4% |
|  | | | | | | | | | | | | | | | |
| **PLM scale** | **Criterion 2 or 3 pathologic biomarkers** | | | **Distribution of AD** | | | | **Distribution of NAD** | | | | **% of AD in each class** | | | |
|  | **Sens.** | **Spe.** | **AUC** | **Class 0** | **Class 1** | **Class 2** | **Class 3** | **Class 0** | **Class 1** | **Class 2** | **Class 3** | **Class 0** | **Class 1** | **Class 2** | **Class 3** |
| Paris-1 | 91.5% | 90.6% | 0.940 | 5.1% | 3.4% | 19.5% | 72.0% | 64.2% | 26.4% | 7.5% | 1.9% | 15,0% | 22,2% | 85,2% | 98,8% |
| Paris-2 | 85.4% | 97.1% | 0.931 | 7.3% | 7.3% | 29.3% | 56.1% | 72.1% | 25.0% | 1.5% | 1.5% | 5,8% | 15,0% | 92,3% | 95,8% |
| Lille-1 | 81.8% | 87.3% | 0.887 | 7.0% | 11.2% | 25.9% | 55.9% | 61.8% | 25.5% | 7.8% | 4.9% | 13,7% | 38,1% | 82,2% | 94,1% |
| Lille-2 | 87.7% | 90.5% | 0.919 | 5.5% | 6.8% | 16.4% | 71.2% | 64.3% | 26.2% | 4.8% | 4.8% | 12,9% | 31,3% | 85,7% | 96,3% |
| Mtp-1 | 77.5% | 89.3% | 0.863 | 9.3% | 13.2% | 20.9% | 56.6% | 56.7% | 32.6% | 4.7% | 6.0% | 9,0% | 19,5% | 73,0% | 84,9% |
| Mtp-2 | 88.0% | 88.4% | 0.933 | 1.4% | 10.6% | 13.4% | 74.6% | 48.3% | 40.1% | 8.2% | 3.4% | 2,7% | 20,3% | 61,3% | 95,5% |
| PLM-1 | 80.3% | 87.6% | 0.883 | 6.9% | 12.8% | 20.3% | 60.0% | 58.4% | 29.2% | 7.3% | 5.1% | 11,1% | 31,6% | 74,5% | 92,5% |
| PLM-2 | 87.1% | 89.1% | 0.924 | 3.5% | 9.4% | 13.3% | 73.8% | 50.2% | 38.9% | 7.8% | 3.1% | 6,5% | 19,4% | 63,0% | 95,9% |

Upper part: the probability of AD (p(AD)) obtained after logistic regression was used with a threshold at 0.5 to calculate the sensitivity (Sens.) and specificity (Spe.) for AD in the different populations (Paris-1,…). The AUCs were also determined from the ROC curves. Four classes based on the values of p(AD) were defined as follows: “class 0”: p(AD) 0 to 0.1; “class 1”: 0.1 to 0.5; “class 2”: 0.5 to 0.9 and “class 3”: 0.9 to 1. The distribution of the AD and NAD populations across the four classes, as well as the % of AD patients in each class was then calculated.

Lower part: the sensitivity (Sens.) and specificity (Spe.) for AD in the different populations (Paris-1,…) were determined based on the criterion that the levels of 2 or 3 biomarkers out of Aβ42, tau and p-tau were pathological (i.e. below the optimal cutoff for Aβ42 and above for tau and p-tau). The PLM scale made of four classes was then defined as such: “class 0”: 0 biomarkers out of 3 pathologic biomarkers: “class 1”: 1 pathologic biomarker, “class 2”: 2 pathologic biomarkers and “class 3”: the 3 biomarkers were pathological. The distribution of the AD and NAD populations across the four classes, as well as the percentage of AD in each class was calculated.
